# A Gene Expression Signature to Predict Nucleotide Excision Repair Defects and Novel Therapeutic Approaches

**DOI:** 10.3390/ijms22095008

**Published:** 2021-05-08

**Authors:** Rongbin Wei, Hui Dai, Jing Zhang, David J. H. Shih, Yulong Liang, Pengfeng Xiao, Daniel J. McGrail, Shiaw-Yih Lin

**Affiliations:** 1 State Key Laboratory of Bioelectronics, National Demonstration Center for Experimental Biomedical Engineering Education, School of Biological Science and Medical Engineering, Southeast University, Nanjing 210096, China; rongbinwei@outlook.com; 2 Department of Systems Biology, The University of Texas MD Anderson Cancer Center, Houston, TX 77030, USA; hdai@mdanderson.org (H.D.); jzhang36@mdanderson.org (J.Z.); DShih@mdanderson.org (D.J.H.S.); yliang7@mdanderson.org (Y.L.); xiaopf@seu.edu.cn (P.X.)

**Keywords:** gene expression signature, nucleotide excision repair defects, breast cancer, cancer treatment, novel therapeutic approaches

## Abstract

Nucleotide excision repair (NER) resolves DNA adducts, such as those caused by ultraviolet light. Deficient NER (dNER) results in a higher mutation rate that can predispose to cancer development and premature ageing phenotypes. Here, we used isogenic dNER model cell lines to establish a gene expression signature that can accurately predict functional NER capacity in both cell lines and patient samples. Critically, none of the identified NER deficient cell lines harbored mutations in any NER genes, suggesting that the prevalence of NER defects may currently be underestimated. Identification of compounds that induce the dNER gene expression signature led to the discovery that NER can be functionally impaired by GSK3 inhibition, leading to synergy when combined with cisplatin treatment. Furthermore, we predicted and validated multiple novel drugs that are synthetically lethal with NER defects using the dNER gene signature as a drug discovery platform. Taken together, our work provides a dynamic predictor of NER function that may be applied for therapeutic stratification as well as development of novel biological insights in human tumors.

## 1. Introduction

The hallmark of human cancer can be classified into six biological capabilities: maintaining proliferative signal, evading growth suppressors, resisting cell death, enabling replicative immortality, inducing angiogenesis, and activating invasion and metastasis. Genome instability has been shown as undertaking these events [1]. To sustain genome integrity and keep high-fidelity genetic message transmission, there is a set of complicated repair machinery in response to DNA damage in cells. Numerous structurally unrelated DNA damages were removed by nucleotide excision repair (NER) using a versatile ‘cut and paste’ mechanism [2]. RNA polymerase II stalling in transcriptional genes was generally caused by massive DNA damages containing ultraviolet light (UV)-induced pyrimidine dimers. The transcription-coupled NER (TC-NER) removes stalled RNA polymerase and repairs these damages, initiating by the Cockayne syndrome proteins CSA and CSB/ERCC6. Once RNA polymerase has been eliminated, the Xeroderma pigmentosum (XP) proteins can catalyze DNA damage repair [3]. The global genome NER (GG-NER) is triggered by Xeroderma pigmentosum complementation group C (XPC) and performs by probing the genome for helix-distorting base lesions [4]. GG-NER deficiency predisposes to cancer development, whereas defective TC-NER results in all kinds of diseases, including ultraviolet radiation-sensitive syndrome and severe premature ageing conditions such as Cockayne syndrome [2].

Breast cancer represents the most common types of tumor diagnosed among women and is responsible for the majority of female cancer-related deaths [5]. Striking histopathological characteristics commonly served as prognostic and predictive biomarkers in clinical therapeutic applications [6]. Nevertheless, there is a challenge to understand breast cancer heterogeneity and precisely predict clinical outcomes only depending on these features [7]. Data derived from genome-wide researches have determined defective DNA repair signatures caused by categorizing mutational types, but the impact of these studies has been diluted by uncertainty regarding the molecular origin and clinical relevance of these signatures [8]. Thus, we propose a hypothesis that defective DDR is involved in cancer response by analyzing molecular status.

The NER pathway involves a large amount of proteins that can recognize, verify, signal, and repair DNA damage [2]. It can be used, as an example, to understand the clinical influence of many DDR processes including cell cycle checkpoint, transcriptional responses, and extensive post-translational modifications [9]. Recent studies have uncovered that a number of genes are involved in NER repair [2]. However, the understanding of the molecular mechanism of defective NER generating by gene mutations is still unclear. Here, a transcriptional profiling-based method was established to systematically distinguish common molecular alterations related to defective NER repair and generate defective NER gene signatures. We found that the dNER gene expression signature predicted loss of NER function in both cell lines and primary patient samples. Leveraging this signature, we further identify multiple novel synthetic lethal therapeutic strategies to directly target NER deficient tumors, as well as novel agents to inhibit dNER as a rational combination to sensitize to current standard chemotherapeutic regimens. Taken together, the dNER gene signature established in our study enables prediction of NER capacity to improve personalized medicine approaches as well as our understanding of the NER pathway.

## 2. Results

### 2.1. Generation and Validation of Isogenic NER Deficient Cell Lines

**Figure 1:**
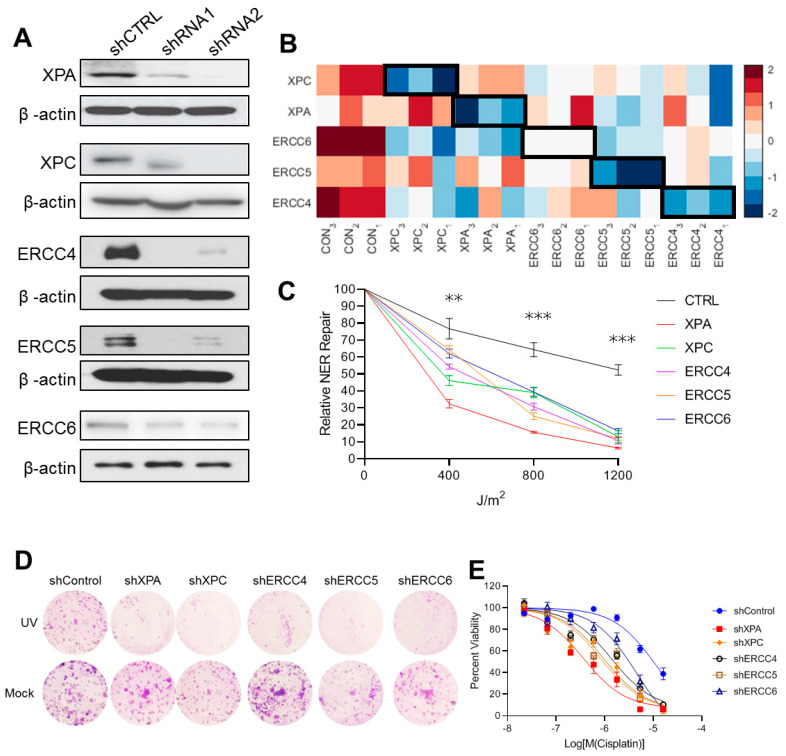
Characterization of isogenic deficient nucleotide excision repair (dNER) model cell lines. (**A**) Western blots demonstrating knockdown of NER genes. (**B**) Heatmap showed the knockdown effect of NER-related genes by RNAseq analysis. (**C**) Measurements of DNA repair capacity (DRC) by fluorescence-based multiplex flow-cytometric host cell reactivation assay (FM-HCR); DNA lesions are introduced into fluorescent reporter plasmids in vitro normalized to a transfection efficiency control. Numbers labeling the plasmids represent the dose (in joules per square meter) of UV radiation. After 48 h incubation, cells were assayed for fluorescence by flow cytometry. ** *p* < 0.01, *** *p* < 0.001. (**D**) Representative colony formation assays with defective and intact breast cancer cell lines. Anchorage-independent colonies were treated with 30 J/m^2^ UV treatment and grown for 7 days before replacing the media for 14 additional days. (**E**) The indicated cancer cell lines were treated with cisplatin for 5 days before assessing cell viability. Each value is relative to the value in the cells treated with vehicle control. Results are shown as mean ± s.e.m. from three independent experiments.

In order to directly probe molecular changes associated with defective NER, we generated isogenic dNER cell lines using non-malignant, genomically-stable, MCF-10A mammary epithelial cells. To induce dNER, we depleted five independent dNER repair genes: XPA, XPC, ERCC4, ERCC5, and ERCC6 (Figure 1A,B). XPA has a key role in coordinating the NER complex owing to its multiple functions in NER repair. XPC serves as a vital DNA damage sensor by stabilizing and assisting the RAD23B, a UV excision repair protein, and centrin 2 (CETN2) in GG-NER machinery [10,11,12]. XPG/ERCC5, a structure-specific endonuclease, either related to transcription Factor II H (TFIIH) or separately, binds to the preincision NER complex [2]. The XPF/ERCC4–ERCC1 heterodimer is directed to the damaged strand by RPA to create an incision 5ʹ to the lesion. CSB/ERCC6 are required for further assembly of the TC-NER machinery, which includes the core NER factors and several TC-NER-specific proteins [13].

To validate that NER was functionally impaired in our model isogenic cell lines, we first analyzed the ability of cells to repair an eGFP plasmid damaged with 400–1200 J/m^2^ of UV light. In this assay, NER capacity is detected as restoration of eGFP expression normalized to a non-damaged transfection control [14]. Across all levels of UV damage analyzed, we found that NER was inhibited in all five dNER cell lines (Figure 1C). To further confirm these results, we next analyzed the ability of isogenic cell line models to survive following exposure to UV light [15] and cisplatin [16]—recovery from both of these requires NER function. While the majority of shCTRL cells were able to recover from exposure to 30 J/m^2^ UV light, as indicated by clonogenic capacity, dNER lines failed to recover from UV damage (Figure 1D). Similar results were obtained following treatment with cisplatin, with all five isogenic dNER lines gaining sensitivity to cisplatin treatment (Figure 1E).

### 2.2. Identification of a Predictive dNER Gene Expression Signature

**Figure 2:**
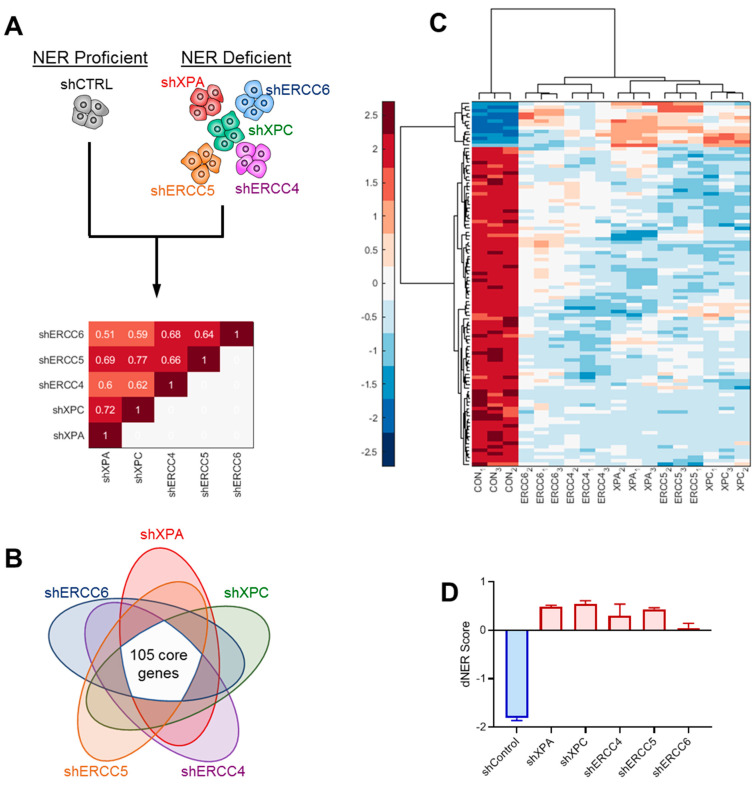
Generation of dNER gene expression signature. (**A**) RNAseq analysis comparing NER proficient shCTRL cells to various NER deficient isogenic cell lines. Correlogram indicates Spearman correlation coefficient for gene expression changes in indicated cell lines relative to shCTRL cells. (**B**) Venn diagram showing 105 core genes differentially regulated with an FDR <0.05 and fold change >1.5 in all five model cell lines selected for dNER gene expression signature. (**C**) Hierarchal clustering using genes from the dNER gene expression signature. (**D**) dNER score in MCF10A knockdown cell lines.

To understand how deficient NER transcriptionally rewires cells, we performed RNA sequencing on all five isogenic dNER cell line models compared with NER proficient shCTRL cells, and found highly correlated transcriptional changes in all five dNER cell lines (R = 0.51–0.77, Figure 2A). To identify core transcriptional changes associated with NER deficiency, we selected genes with an absolute fold change greater than 1.5 and false discovery rate less than 0.05 in all five cell lines, yielding a 105 gene transcriptional signature (Figure 2B, Table S1). This dNER gene expression signature clearly divided model cell lines both by hierarchal clustering (Figure 2C) and by calculation of a gene expression score (Figure 2D).

### 2.3. Functional Prediction of dNER in Breast Cancer Cell Lines

**Figure 3:**
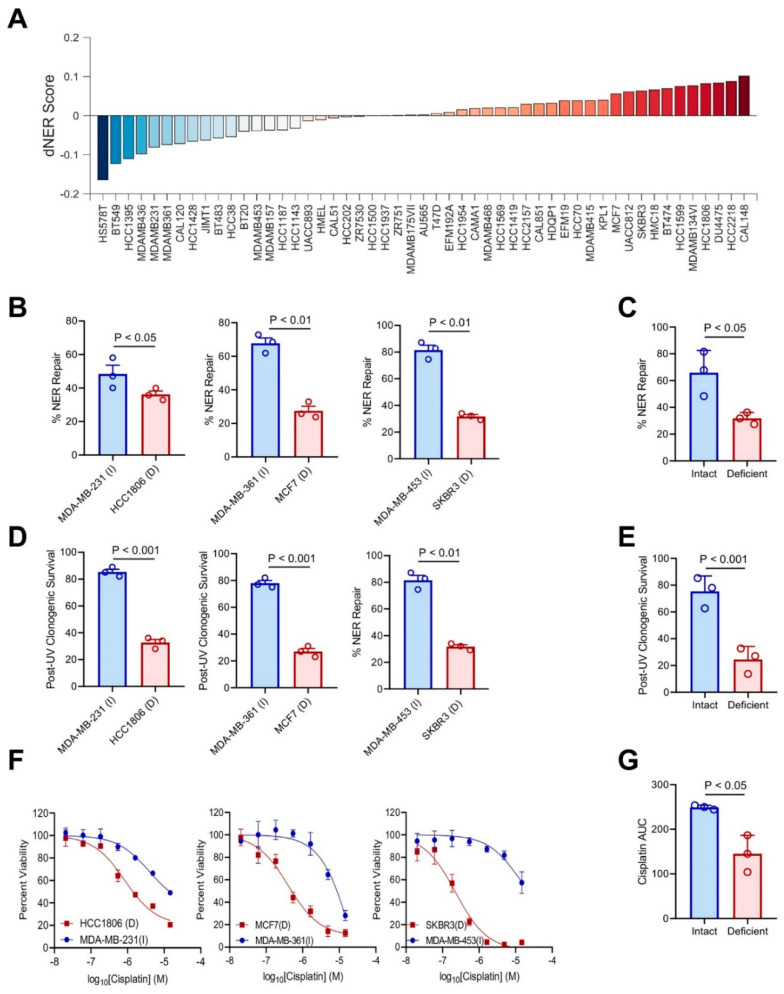
The dNER gene expression signature predicts NER function in breast cancer cell lines. (**A**) dNER score in a panel of breast cancer cell lines. (**B**) NER functional reporter assay as described in Figure 1C performed in an NER deficient (D) and NER intact (I) cell line pair from each breast cancer subtype: basal/triple negative HCC1806 (D) and MDA-MB-231 (I), luminal MCF7 (D) and MDA-MB-361 (I), and HER2 SKBR3 (D) and MDA-MB-453 (I). Each dot represents an independent biological replicate. (**C**) Combined data from 3B, with each dot representing the average of an individual cell line. Paired *t*-test. (**D**) Clonogenic capacity following UV radiation at 30 J/m^2^ compared with mock treatment in cell lines from (B). Each dot represents an independent biological replicate. (**E**) Combined data from (D), with each dot representing the average of an individual cell line. Paired *t*-test. (**F**) Sensitivity of cell lines from (B) to cisplatin treatment. Data plotted as mean+/-s.e.m. of three independent biological replicates. (**G**) Combined data from (F), with each dot representing the area under the viability curve of an individual cell line. Paired *t*-test.

In order to assess the functional predictive capacity of our dNER gene expression signature, we calculated dNER scores across a panel of breast cancer cell lines (Figure 3A). From each breast cancer subtype, we selected an NER deficient (D) and NER intact (I) cell line pair for functional analysis: basal/triple negative HCC1806 (D) and MDA-MB-231 (I), luminal MCF7 (D) and MDA-MB-361 (I), and HER2 SKBR3 (D) and MDA-MB-453 (I). Using the NER fluorescence reporter assay, we found NER activity was significantly impaired in predicted NER deficient lines in all three cell line pairs (Figure 3B,C). This impaired NER activity was further validated by dNER cell lines showing impaired recovery from UV exposure (Figure 3D,E and Figure S1) as well as increased sensitivity to cisplatin (Figure 3F,G). Taken together, these results suggest that our dNER transcriptional signature can accurately predict NER functional deficiencies in breast cancer cell lines.

### 2.4. Activity of dNER Signature in Primary Patient Samples

**Figure 4:**
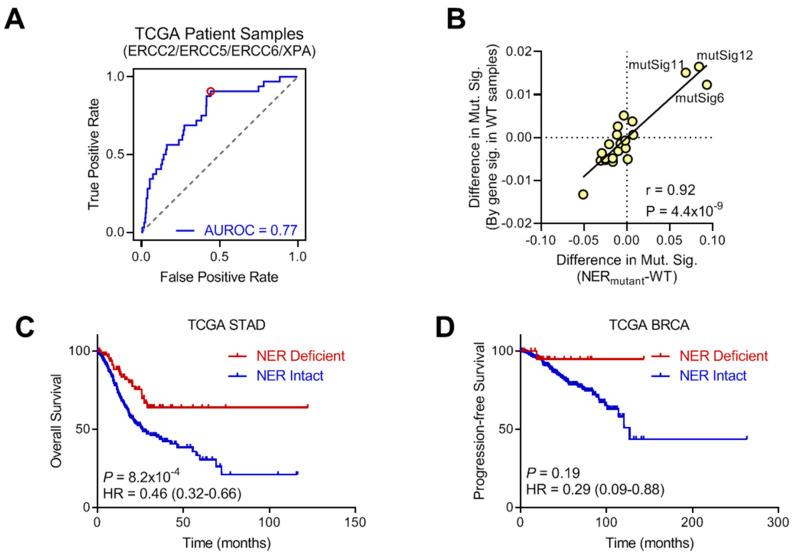
Prediction of NER function in primary patient tumors. (**A**) ROC curve for prediction of gastric tumors with mutation in NER genes by dNER gene expression score. (**B**) Correlation between alterations in mutational signatures in either tumors with mutations in NER genes (*x*-axis) compared with tumors with NER mutations predicted to be NER deficient by gene expression (*y*-axis). Pearson correlation coefficient. (**C**) Overall survival of patients with gastric cancer, stratified by predicted NER function. Log-rank test. (**D**) Progression-free survival of patients with breast cancer, stratified by predicted NER function. Log-rank test.

To further validate our signature, we next sought to determine if our dNER signature was predictive in vivo using primary patient samples from The Cancer Genome Atlas (TCGA). First, we assessed if the dNER signature score could predict tumors with mutations in critical NER genes. As breast tumors lacked sufficient tumors with mutated NER genes for this analysis, we focused on gastric cancer, where 8.6% of tumors had mutations in an NER gene. As illustrated by the receiver operating characteristic (ROC) curve in Figure 4A, we observed robust prediction of NER deficient tumors with an area under the ROC curve (AUROC) value of 0.77. At the optimal threshold determined by Youden’s statistic, this corresponded to a 90.6% sensitivity and 44.2% false positive rate, though it is unclear how many theoretical false positives are actually NER defective, but lack any identified mutations in NER genes. As NER defects will lead to increased mutagenesis, we hypothesized that, if theoretical false positive tumors are actually NER defective, they would exhibit similar mutational characteristics as tumors with mutations in NER genes. To evaluate this, we next analyzed the mutagenic processes operative within tumors as quantified by mutational signatures [17] in three distinct groups: (1) “gold-standard” dNER tumors with mutations in NER genes; (2) theoretical false positive dNER tumors predicted to be dNER, but lacking any mutations in NER genes; and (3) tumors predicted to be NER proficient by gene expression and lacking mutations in any NER genes. Comparing changes in mutational signatures between group 1 “gold-standard” dNER tumors and group 2 predicted dNER tumors relative to group 3 NER proficient tumors revealed highly concordant alterations in mutational processes (Figure 4B), indicating that our dNER transcriptional signature is likely predicting NER defects in primary patient tumors. As NER deficiencies sensitize to chemotherapeutics such as cisplatin, we further hypothesized patients with dNER tumors would exhibit better prognosis than those with NER proficient tumors. Consistent with our hypothesis, patients with gastric cancer exhibited improved overall survival if their tumors were predicted to be dNER (*p* = 8.2 × 10^−4^, hazard ratio = 0.46, Figure 4C). Although breast tumors did not harbor significant mutations in dNER genes, the subset of patients with breast cancer with predicted dNER tumors exhibited improved prognosis as well (Figure 4D). These results indicate that our dNER gene expression signature can predict NER defects in primary patient samples.

### 2.5. Prediction of Compounds to Inhibit NER

**Figure 5:**
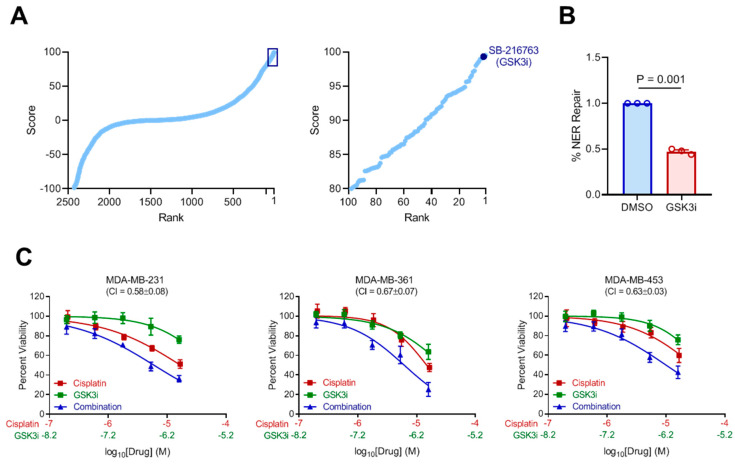
Inhibition of glycogen synthase kinase (GSK) impairs NER function. (**A**) Prediction of compounds that induce the dNER gene expression signature using LINCS identifies GSK inhibition as a top candidate. (**B**) NER functional assay as described in 1C following treatment with 100 nM GSK3 inhibitor SB-216763. (**C**) The indicated cancer cell lines were treated with DMSO, cisplatin, GSK3bi, or a combination thereof for 5 days before assessing cell viability. Results are shown as mean ± s.e.m. from three independent experiments.

Given that the dNER gene signature can functionally link transcriptional changes to NER repair deficiency, we asked whether we could identify agents that would induce the dNER gene signature and, thereby, induce sensitivity of cancer cells to DNA damage-inducing treatment such as cisplatin. To this end, we used the Library of Integrated Network-based Cellular Signatures (LINCS), a catalogue of transcriptional alterations induced by treatment with various drugs or other perturbations [18]. We looked for compounds that induced the dNER transcriptional program. Among the top candidates identified was an inhibitor of glycogen synthase kinase 3 (GSK3) (Figure 5A). As GSK has been shown to have tumor-promoting roles in diverse cancers, such as bladder cancer [19], osteosarcoma [20], leukemia [21], and glioblastoma [22], we selected it for further study based on the hypothesis that it may both induce NER defects leading to cisplatin and provide independent tumor killing effects. Initial testing of NER function using the flow reporter assay indicated that inhibition of GSK3 significantly inhibited NER repair (Figure 5B). Combination of GSK3 inhibition with cisplatin led to synergistic activity in three independent NER proficient cell lines (Figure 5C), suggesting it may present a novel combination therapy that could benefit patients.

### 2.6. Identification of Novel Compounds to Treat dNER Tumors

**Figure 6:**
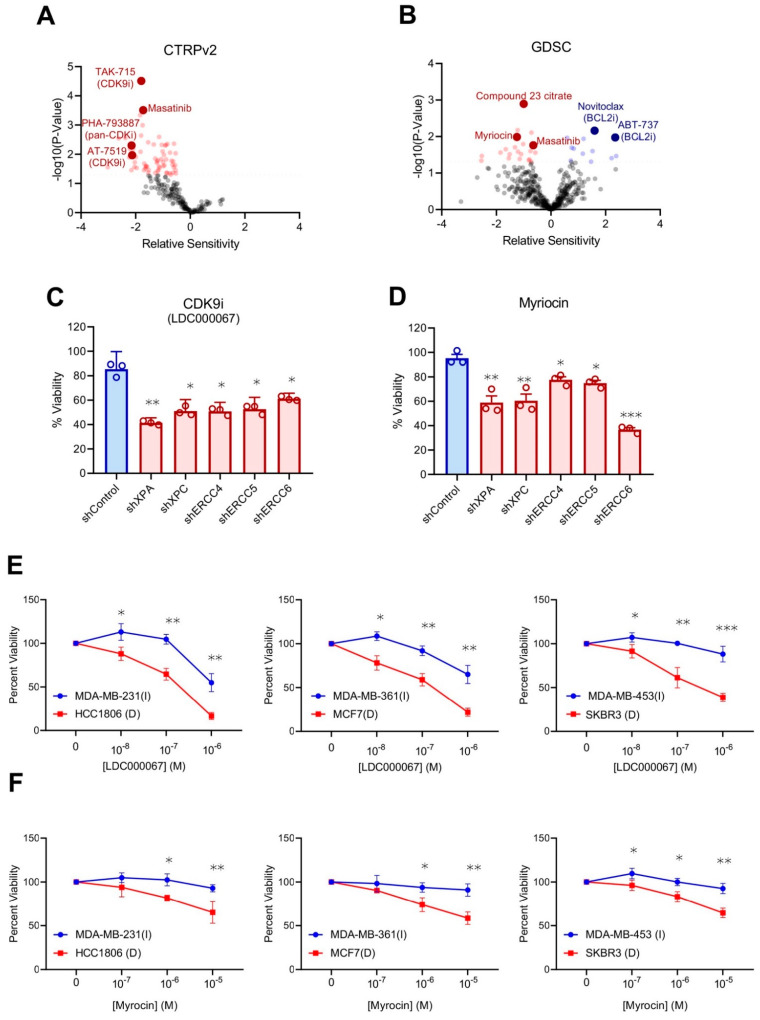
Discovery of novel therapeutic strategies for dNER tumors. (**A**) Prediction of drug responsiveness by dNER gene-expression signature in CTRPv2 cancer cell lines, where negative values indicate increased predicted sensitivity. (**B**) Prediction of drug responsiveness by dNER gene-expression signature in GDSC cancer cell lines, where negative values indicate increased predicted sensitivity. (**C**) Viability of MCF10A isogenic model cell lines treated with 1 μM CDK9 inhibitor LDC000067 for 5 days. Each value is relative to the value in the cells treated with vehicle control. Results are shown as mean ± s.e.m. from three independent experiments. (**D**) Viability of MCF10A isogenic model cell lines treated with 1 μM Myriocin for 5 days. Each value is relative to the value in the cells treated with vehicle control. Results are shown as mean ± s.e.m. from three independent experiments. (**E**) NER deficient (D) and intact (I) cell lines from 3B were treated with the indicated dosages of CDK9 inhibitor LDC000067 for 5 days. Each value is relative to the value in the cells treated with vehicle control. Results are shown as mean ± s.e.m. from three independent experiments. (**F**) NER deficient (D) and intact (I) cell lines from 3B were treated with the indicated dosages of Myriocin for 5 days. Each value is relative to the value in the cells treated with vehicle control. Results are shown as mean ± s.e.m. from three independent experiments. * *p* < 0.05, ** *p* < 0.01, *** *p* < 0.001.

Although dNER tumors exhibit enhanced responses to platinum and other chemotherapies, these therapeutic modalities generally cause undesirable side effects, lessening patient quality of life. We hypothesized the NER defects may lead to novel synthetically lethal therapeutic approaches that would enhance the therapeutic index, resulting in decreased side effects and improved quality of life, much in the way that PARP inhibitors have done for patients with tumors that have deficient homologous recombination DNA repair. Using our previously established algorithm to predict novel therapeutic vulnerabilities [23,24], we identified a series of compounds that may target dNER cells from both CTRPv2 (Figure 6A) and GDSC (Figure 6B) drug sensitivity databases. From CTRPv2, we detected multiple inhibitors of CDK9 predicted to preferentially kill dNER tumor cells. We evaluated this prediction using our isogenic dNER cell lines with an orthogonal CDK9 inhibitor, and found that, while NER proficient shControl cells exhibited minimal loss of viability upon CDK9 inhibition, the drug was toxic to dNER cell lines (Figure 6C). Likewise, evaluating myriocin from the GDSC sensitivity library produced similar results with increased sensitivity in dNER lines (Figure 6D). Finally, we confirmed that both predicted compounds preferentially killed tumor cell lines harboring endogenous NER deficiencies (Figure 6E,F), suggesting these compounds may have therapeutic potential for the treatment of dNER tumors.

## 3. Discussion

Eukaryotic cells are involved in repairing many kinds of DNA lesions. Among the well-known DNA repair processes in humans is NER repair, which specifically can protect against mutations caused indirectly by environmental carcinogens, and then maintain genomic integrity and prevent tumorigenesis. NER repair can remove various structurally unrelated DNA damages such as UV-induced damage. Extreme cancer proneness (xeroderma pigmentosum) or dramatic premature aging (Cockayne syndrome) caused by germline/hereditary NER deficiency illustrate how the importance of NER repair [25]. Owing to the complexity of NER repair, there is an enormous challenge to identify dysfunctional NER repair in human tumors. Here, we established a functional network view of the consequences of defective NER using gene expression profiling. Our studies showed that NER repair components were not independent, but a functional network, involved in cellular integrated NER repair capability. The dNER model can allow us to dynamically monitor the NER repair status by simultaneously considering hundreds of genes and, thereby, allow to validate the functional NER deficiency in a given cellular state independent of an underlying mechanism.

Cisplatin primarily causes DNA lesions by forming intra-strand crosslinks, with the formation of guanine–platinum–guanine and guanine–platinum–adenine adducts [26]. Intra-strand crosslinks caused by cisplatin accounted for 90% of lesions, with additional lesions rarely forming guanine–platinum–guanine inter-strand crosslinks [26]. Nucleotide excision repair (NER) is responsible for the repair of intra-strand DNA crosslinks, while it is considered as single-strand DNA (ssDNA) repair mechanism that is defective in clinical patients with xeroderma pigmentosum [27]. Thus, NER defects may sensitize to cisplatin or other chemotherapeutic modalities. Indeed, we found knockdown genes in an NER repair pathway induced cisplatin sensitivity in our isogenic cell line models as well as cell lines predicted to be NER deficient. Consistent with our cell line results, in gastric cancer patients predominately treated with a platinum-containing regimen, patients with dNER tumors showed improved prognosis compared with predicted NER intact counterparts, with consistent results observed in breast cancer. While both NER and homologous recombination (HR) defects can contribute to chemosensitivity [26,28], additional functional repair assays directly probing the ability of cells to perform NER indicate that our transcriptional signature can predict functional NER defects. Moreover, by identifying drugs that can induce our dNER signature, we found that GSK3 inhibition can sensitize to treatment with cisplatin. We selected GSK3 from among the top candidates as it has been shown to be activated in cancer types including bladder cancer [19], osteosarcoma [20], leukemia [21], and glioblastoma [22], which may provide for monotherapy efficacy as well. As predicted, we found that inhibition of GSK3 both inhibited NER repair capacity and synergized with cisplatin treatment. This synergy is consistent with other works showing that inducing of dNER by targeted inhibition of XPA can sensitize to cisplatin to broaden the usefulness of this chemotherapeutic agent [16].

While dNER tumors have shown sensitivity to platinum agents, we next ask if dNER tumors may have novel synthetic lethal therapeutic vulnerabilities that would not necessitate such strong systemic side effects and improve patient quality of life, much as targeting homologous recombination with PARP inhibitors has down in BRCA1/2 mutant tumors. We identified multiple candidate compounds to target dNER, notably Myriocin/M1177 and CDK9 inhibitors. Myriocin inhibits sphingolipid biosynthesis, which is involved in cell biological processes, including growth regulation, cell migration, adhesion, apoptosis, senescence, and inflammatory responses [29]. Targeting sphingolipid metabolism to activate pro-cell death ceramide signaling and/or inhibit pro-survival sphingosine-1-phosphate (S1P) signaling is done using genetic, molecular, immunological, or pharmacological tools [30]. Myriocin has also been shown to exacerbate consequences of proteotoxic stress [31], which is prevalent in cancer cells [32]. Cyclin-dependent kinase 9 (CDK9), an important regulator of transcriptional elongation, is a promising target for cancer therapy, particularly for cancers driven by transcriptional dysregulation [33]. Previous studies reported that activity of CDK9 was involved in maintaining a high expression level of MDM4 in human cells, and drugs targeting CDK9 might restore p53 tumor suppressor function in malignancies overexpressing MDM4 [34]. Moreover, CDK9 is a promising prognostic marker and therapeutic target in cancers [35], including activity castration-resistant prostate cancers (CRPCs) models [36]. Other studies have shown a therapeutic benefit of combined cisplatin with a CDK9 inhibition, which could be particularly potent in dNER tumors [37]. Future studies probing these novel synthetic lethal interactions in dNER tumors may shed further insight into NER biology.

Thus, taken together, our work identifies a novel transcriptional signature that can predict defects in nucleotide excision repair across patients and cell lines. We leverage this signature to identify novel compounds that may synergize with current platinum-based chemotherapeutic regimens, as well as novel synthetic lethal interactions with loss of NER function. These findings will have implications both for personalized cancer therapies as well as for gleaning a further understanding of NER repair.

## 4. Materials and Methods

### 4.1. Cell Culture and Reagents 

MCF10A cell lines was obtained from American type culture collection (ATCC) and were grown in DMEM/F12 Ham’s Mixture medium supplemented with 5% Equine Serum, 20 ng/mL epidermal growth factor, 10 μg/mL insulin, 0.5 mg/mL hydrocortisone, 100 ng/mL cholera toxin, 100 μg/mL streptomycin, and 100 units/mL penicillin. MCF7, BT549, MDA-MB-468, MDA-MB-231, HCC1806, MDA-MB-461, MDA-MB-453, and SKRB3 cell lines were purchased from ATCC and cultured in appropriate mediums supplemented with 10% FBS, 100 μg/mL streptomycin, and 100 units/mL penicillin according to ATCC guidelines respectively. XPA (D9U5U) Rabbit monoclonal (CST #14607), XPC Polyclonal Antibody (CST#12701), XPF/ERCC4 Rabbit Polyclonal Antibody (Bethyl A301-315A), ERCC5/XPG Rabbit Polyclonal Antibody (Bethyl A301-485A), and ERCC6/CSB Rabbit Polyclonal Antibody (Proteintech Catalog number: 24291-1-AP) were purchased from CST and Bethyl Laboratories company. CDK9 inhibitors LDC000067 (Catalog number: S7461), Myriocin from *Mycelia sterilia* (M1177) (CAS Number 35891-70-4), and SB-216763/GSK inhibitors (Catalog number: S3442) were purchased from Sigma.

### 4.2. Lentiviral Infection and Plasmid Transfection

Mission shRNA lentiviral particles, namely, clones TRCN0000083196 (XPA), TRCN0000307193 (XPC), TRCN0000507788 (ERCC5), TRCN0000016774 (ERCC6), TRCN0000078583 (ERCC4), and mission shRNA non-target control transduction particles, were purchased from Sigma Aldrich. Day 1: MCF-10A cells were counted and then seeded 5 × 10^4^ cells in a six-well plate with fresh media, and each lentiviral construct and control groups were used in triplicate wells. Day 2: half of the old media were replaced by fresh media and 2–15 μL individual lentiviral virus (Sigma) targeting XPA, XPC, ERCC4, ERCC5, ERCC6 was added to each well with 8 μg/mL Hexadimethrine bromide. Then, the six-well plate was swirled lightly to mix and incubated in a humidified 5% CO_2_ incubator at 37 °C. Day 3: we removed the media containing lentiviral particles from the six-well plate, and then added fresh media to a volume of 2 mL to each well. Day 4: we added fresh media with 2 μg/mL puromycin. Day 5 and on: fresh puromycin media were replaced every 3~4 days until the formation of resistant colonies. Finally, the stable resistant cell lines were identified.

### 4.3. Western Blot Analysis

Cells were lysed in urea buffer (8 M urea, 150 mM β-mercaptoethanol, and 50 mM Tris/HCl, pH 7.5), and cleared by centrifugation (14,000× *g* rcf for 15 min at 4 °C). Protein concentration was determined using the bicinchoninic acid assay (BCA). Proteins were separated by gel electrophoresis and transferred to polyvinylidene difluoride membranes, and then probed with the desired antibodies.

### 4.4. NER Repair Analysis

We tested NER repair capacity using fluorescence-based multiplex flow-cytometric host cell reactivation assay (FM-HCR) [14]. In brief, EGFP-C1 (Addgene plasmid # 46956) was irradiated with the indicated dosage of UV light (400–1200 J/m^2^), introducing DNA lesions that block the ability for the plasmid to be transcribed. In NER proficient cells, the UV-induced lesions in EGFP-C1 can be repaired, restoring EGFP expression. Cells were co-transfected with irradiated EGFP-C1 to monitor NER repair and non-irradiated pCMV-tdTomato (Addgene plasmid #30530) as an endogenous transfection control. Transfections were performed using Lipofectamine 3000 per the manufacturer’s instructions. After 72 h, cells were analyzed by flow cytometry. NER repair was determined as the percentage of tdTomato^+^ cells with restored EGFP expression using FlowJo.

### 4.5. Colony Formation Assay

Cells were seeded into six-well plates with fresh media overnight at an appropriate density; the following day, the cells were treated with 30 J UV or the indicated concentrations of drugs. The media was replaced with fresh media every 3~4 days to allow colonies to form. Then, cells were fixed in cold methanol and stained with 0.25% crystal violet at room temperature for 30 min. After washing three times with wash buffer (phosphate-buffered saline, PBS), colonies were counted manually or quantified by ImageJ.

### 4.6. Cell Proliferation Assay

Cells were seeded into a 96-well plate containing 100 µL/well of cell culture medium and incubated overnight before initiation of drug treatment. Seven days later, we added 20 ul of PrestoBlue substrate (2 mg/mL) to each well and incubated them at 37 °C incubator for 2~4 h; viability was detected using a fluorescent plate reader with 560 nm excitation. After subtraction of background, the cell viability was calculated relative to vehicle control (DMSO) cells.

### 4.7. Drug Combination Studies

For drug combination studies, the results were gained using the PrestoBlue™ Cell Viability assays in triplicate. The combination index (CI) was analyzed following the User’s Guide of the CompuSyn software using the combination index (CI)-isobologram equation, which indicated the all dose–effect curve [38]. The equation allowed researchers to quantitatively define drug interactions, where CI < 1 indicated synergism, CI = 1 indicated additive, and CI > 1 indicated antagonism.

### 4.8. RNAseq Analysis and Signature Generation

RNA was isolated from three independent biological replicates using a QIAgen RNeasy kit and sequenced by NovoGene. The sequencing samples were performed 20 M raw reads/sample. Library type: 250~300 bp was inserted into cDNA library, and we performed double-stranded cDNA sequencing and rRNA depletion. RNAseq FASTQ files were quantified using kallisto v0.44.0 [39] and are available in Table S1. The dNER gene expression signature (Table S2) was taken as genes with FDR <0.05 (as assessed by Storey method) and absolute fold change values >1.5. Coefficients were determined as the average fold change value from all five cell lines. Signature score was calculated by summing over the product of the gene coefficient and z-normalized log-transformed gene expression value, normalized to the sum of the absolute value of the gene coefficients. Hierarchical clustering was performed using Ward linkage.

### 4.9. TCGA Analysis

All TCGA data were downloaded using the TCGA data portal (https://portal.gdc.cancer.gov/) from the Pan-Caner Atlas release (April 2018). Mutation signature scores were acquired from Knijnenburg et al. [40]. The optimal threshold to stratify tumors by NER status was determined by Youden’s index with bootstrapped confidence intervals. Survival was assessed by log-rank test.

### 4.10. Prediction of Drugs to Inhibit NER and Novel dNER-Targeting Drugs

To identify drugs that may inhibit NER, we utilized the LINCS ConnectivityMap data to evaluate if any of over 80,000 perturbagens evaluated may induce our dNER signature. All analysis was performed on clue.io using default parameters. We used our previously established algorithm to predict novel therapeutic vulnerabilities in dNER tumor cells [23,24]. In brief, breast cancer cell lines were divided by gene expression-predicted NER status by dNER score, classifying dNER as the upper quartile of dNER scores. Matched drug sensitivity data acquired from either CTRPv2 [41] or GDSC [42] were then used to predict drugs that preferentially killed dNER tumor cells. Gene expression data were acquired from the Cancer Cell Line Encyclopedia [43].

### 4.11. Statistics

Unless otherwise noted, all experiments were performed in biological triplicates and statistical significance assessed by Student’s *t*-test. Survival was assessed by log-rank test.

## 5. Conclusions

In this work, we used isogenic model cell lines to identify a gene expression signature for nucleotide excision repair defects (dNER) from core transcriptional changes associated with loss of NER function. We validated that our dNER signature can functionally predict NER defects both in cell lines using functional assays, as well as in patient samples using known mutations in NER genes and survival readouts. Critically, the dNER cell lines we identified lacked mutations in NER genes, suggesting the prevalence of NER may be underestimated in cancer. We further leverage this signature to identify and validate novel synthetic lethal drugs with NER deficiency, as well as potential agents to synergize with current chemotherapeutic treatment strategies by inducing NER defects. We feel the implications of this work, for both the fundamental study of NER as well as advancing personalized medicine approaches.

## Data Availability

All data are available in Supplementary Tables or are previously published and publically accessible. The RNAseq data were deposited in GEO database and accession numbers is GSE168861.

## Supplementary Materials

The following are available online at https://www.mdpi.com/article/10.3390/ijms22095008/s1.
