# Supplementary material for: A Gene Expression Signature to Predict Nucleotide Excision Repair Defects and Novel Therapeutic Approaches
Source: Int J Mol Sci. 2021 May 8;22(9):5008. doi: 10.3390/ijms22095008 (PMC8125907; doi:10.3390/ijms22095008)
Supplement: Supplementary file 1 [file ijms-22-05008-s001.zip › ijms-1185729-supplementary/ijms-1185729-suppl- for final/ijms-1185729-suppl-figure.pdf]

International Journal of
Molecular Sciences

MDPI

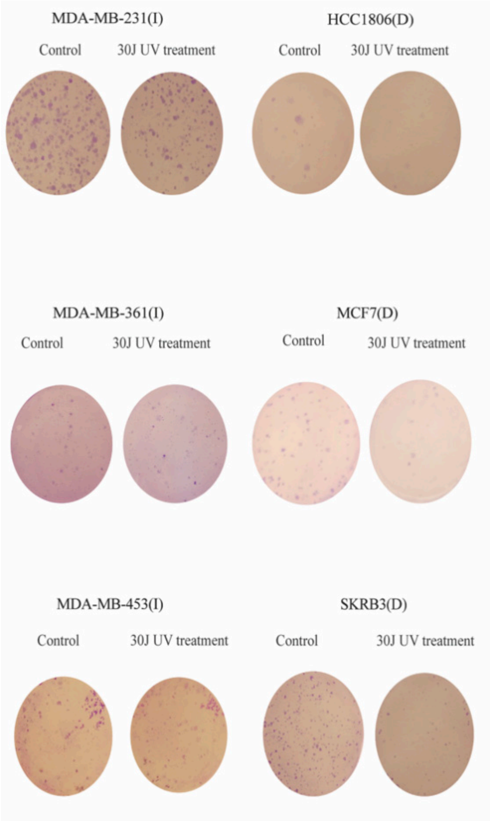

Figure S1. Representative colony formation assays with defective and intact breast cancer cell lines. Anchorage-independent colonies were treated with 30 J UV/m^2 treatment or cisplatin and grown for 7 days before replacing the media for 14 additional days. Results are shown as mean ± s.d. from three independent assays.
